# Cultural alienation and psychological well-being in Mongolian pre-college students: a person-centered profiling study

**DOI:** 10.3389/fpsyg.2025.1730675

**Published:** 2026-01-05

**Authors:** Junying Liu

**Affiliations:** School of Educational Sciences, Baotou Teachers’ College, Inner Mongolia University of Science & Technology, Baotou, China

**Keywords:** cultural alienation, cultural loneliness, Inner Mongolia, minority education, pre-college students, psychological adaptation

## Abstract

**Introduction:**

As educational institutions worldwide strive to support minority students’ academic success and psychological well-being, understanding the complex dynamics of cultural alienation becomes increasingly crucial. This study examines how cultural alienation—including cultural loneliness, separation, disharmony, and perceived discrimination—influences the psychological adaptation of Mongolian minority pre-college students in Inner Mongolia, where institutional practices and social dynamics predominantly reflect Han Chinese cultural norms.

**Methods:**

A sample of 73 participants completed the Symptom Checklist–90 (SCL–90) and a Cultural Alienation Scale developed for minority-focused bridging programs.

**Results:**

Students reported moderate levels of cultural alienation (*M* = 2.40, *SD* = 0.48). Analysis revealed significant associations between overall alienation and psychological distress (*r* = 0.446, *p* < 0.001), particularly with obsessive-compulsive tendencies (*r* = 0.457, *p* < 0.001) and paranoid ideation (*r* = 0.426, *p* < 0.001). Multiple regression analyses identified cultural loneliness as the strongest predictor of psychological symptoms across several domains, notably depression and anxiety, consistently surpassing the effects of other alienation dimensions (*R*^²^ = 0.347 for Depression, *p* < 0.001). Using k-means clustering, we identified two distinct groups: a high-alienation cluster (*n* = 31) reporting substantially greater psychological distress (*M* = 1.60) compared to their low-alienation peers (*M* = 1.27), *F*(1, 71) = 11.57, *p* < 0.001, η^²^ = 0.14.

**Discussion:**

These findings highlight cultural loneliness as a critical mechanism in minority students’ psychological adaptation, providing empirical support for belongingness theory within cross-cultural educational contexts. By demonstrating that social connection deficits, rather than cultural separation or perceived discrimination, most strongly predict psychological distress, this study extends acculturation theory to emphasize the primacy of relational belonging in cultural transitions. These insights advance broader cultural psychology research on minority adaptation while offering practical implications for developing targeted support systems in minority education programs.

## Introduction

Higher education represents a critical juncture where students not only master disciplinary content but also develop social, emotional, and cultural competencies. Growing attention has been directed toward the psychological and behavioral challenges faced by diverse student populations, including students with learning disabilities (Al-Shekaili et al., 2025) and those from minority backgrounds who must navigate distinctive cultural and linguistic contexts. Within China, minority pre-college students typically enter specialized bridging programs designed to aid their transition into mainstream universities where instruction is predominantly delivered in Mandarin. These programs aim to facilitate academic integration through intensive language training and cultural acclimation (Liu, 2023). However, despite official policies encouraging ethnic inclusion and bilingual education, the reality on campus often reveals more complex challenges (Rong, 2007), with cultural, linguistic, and social disparities sometimes fueling a sense of estrangement or inadequate integration.

Located in northern China, Inner Mongolia presents a distinctive educational landscape where minority students from Mongolian backgrounds pursue higher education through government-supported preparatory programs. These initiatives serve as essential gateways for students transitioning from Mongolian-medium secondary education to Chinese-medium university instruction. The programs provide comprehensive academic support, including intensive language training, subject-specific preparation, and cultural orientation (Liu, 2023). However, the transition extends far beyond mere academic adaptation. Students encounter unfamiliar social norms, different pedagogical traditions, and new expectations for peer and faculty interactions. The simultaneous navigation of these academic, linguistic, and sociocultural challenges often creates substantial psychological pressure for students attempting to maintain their cultural identity while adapting to the mainstream university environment (Makarova and Birman, 2015). Pre-college students may be particularly vulnerable to cultural alienation during this critical transition. Unlike senior university students who have established coping strategies and social support networks, pre-college students must simultaneously navigate academic, linguistic, and social challenges without these protective resources. For many Mongolian students, this program represents their first sustained immersion in a Han-dominant institutional environment, and the condensed timeframe of preparatory programs creates additional pressure to adapt rapidly.

The process of cross-cultural adaptation in this context involves complex cognitive, behavioral, and emotional adjustments (Berry and Sam, 1997; Ward et al., 2020). While these theoretical frameworks originated from studies of international migration, they offer valuable insights for understanding the experiences of minority students who, despite remaining within national borders, enter educational environments that function as distinct cultural spaces (Berry, 2019). Students must develop new communication strategies, adjust their learning approaches, and reconstruct their social networks in an environment that operates under different cultural assumptions. Their responses to these challenges vary significantly: some successfully integrate elements of both cultural contexts, others maintain strong connections to their heritage culture while minimally engaging with the majority environment, and some experience profound cultural alienation when institutional support proves insufficient or ineffective (Sun et al., 2024; Syed et al., 2011). These varying adaptation patterns align with Berry and Sam’s (1997) acculturation model and belongingness theory (Baumeister and Leary, 1995), which emphasize that successful cultural adaptation requires both practical competencies and meaningful interpersonal connections. The absence of such essential social bonds may intensify cultural alienation and increase psychological vulnerability.

Research examining minority students in Chinese higher education has predominantly focused on language proficiency and academic performance as primary indicators of successful adaptation (Carter et al., 2013). While these metrics carry obvious significance, they may obscure underlying psychosocial challenges that persist despite satisfactory academic achievement (Carter et al., 2013). Existing investigations of mental health typically address isolated concerns such as linguistic stress or discriminatory experiences, yet this narrow focus potentially overlooks other crucial elements of cultural adaptation—particularly the subjective sense of isolation or perceived incompatibilities with the institutional environment (Carter, 1999). Moreover, research specifically examining minority pre-college curricula remains limited, leaving questions about the psychological impact of this critical transitional period largely unexplored.

Cultural alienation represents a multifaceted construct central to understanding minority students’ experiences, characterized by individuals’ perceived detachment from both surrounding and heritage cultures (Sam and Berry, 2010). Unlike acculturative stress, which emphasizes the psychological strain during cultural adaptation (Berry, 2006), cultural alienation captures individuals’ subjective sense of detachment from cultural contexts—a state that may persist even after initial adaptation challenges have been addressed. This alienation manifests through four interconnected dimensions: cultural loneliness, cultural separation, sense of disharmony, and perceived discrimination (Xi and Dong, 2005). Cultural loneliness reflects profound subjective isolation and absence of meaningful social bonds, frequently associated with elevated anxiety and depressive tendencies (Hawkley and Capitanio, 2015). Recent cross-cultural research has similarly demonstrated that loneliness significantly undermines psychological well-being among international students navigating unfamiliar cultural environments (Ashilah and Edy, 2025). Cultural separation indicates limited engagement with the majority culture, functioning either as an adaptive strategy preserving in-group identity or as an isolating stance that compounds stress, contingent upon existing social support networks (Ward and Kennedy, 1999). Sense of disharmony captures perceived incompatibility between personal and institutional cultural norms, potentially generating frustration when campus environments appear fundamentally misaligned with individual values (Searle and Ward, 1990). Perceived discrimination encompasses experiences of marginalization by the dominant group (Pascoe and Smart Richman, 2009).

These dimensions of cultural alienation demonstrate complex patterns of independence and interaction in their influence on psychological well-being. Some students primarily experience a single dimension, such as profound loneliness, while others confront multiple concurrent challenges. For instance, loneliness alone may precipitate anxiety or depressive symptoms when students lack meaningful connections in their new environment. Alternatively, the combination of high separation tendencies and perceived discrimination might manifest as interpersonal hostility or heightened stress vulnerability (Baumgartner and Susser, 2013). Research has yielded divergent findings regarding these relationships: some evidence suggests that pervasive perceived discrimination significantly undermines psychological well-being (Wang and Mallinckrodt, 2006), while other investigations identify cultural loneliness as the primary mechanism driving negative affect (Cacioppo and Hawkley, 2009). Such variations underscore the importance of examining alienation’s constituent elements both independently and in combination. Students experiencing severe relationship deficits may exhibit different psychological patterns compared to those who perceive fundamental incompatibilities with campus norms, or those who feel targeted due to their ethnic identity. Given varying levels of cultural integration support across bridging programs, clarifying the relative influence of these dimensions emerges as a critical empirical question for developing targeted interventions.

Current methodological approaches to studying cultural alienation warrant expansion beyond traditional variable-centered analyses (Howard and Hoffman, 2018). Student subgroups exhibiting concurrent high levels of cultural loneliness and perceived discrimination, for example, may demonstrate markedly different mental health profiles compared to those reporting moderate disharmony but minimal loneliness. Even students experiencing mild separation might demonstrate effective coping when supported by networks that validate their heritage culture. These distinctions hold particular relevance for minority-oriented bridging programs, where standardized interventions may inadequately address the specific needs of students experiencing distinct configurations of alienation. The identification of such nuanced profiles extends beyond methodological refinement; it represents a conceptual shift toward person-centered approaches in educational psychology, acknowledging that minority students require differentiated forms of psychosocial support based on their unique alienation patterns.

Based on these theoretical and empirical considerations, the present study examines the relationship between cultural alienation and mental health among Inner Mongolian minority pre-college students through multiple analytical lenses. We hypothesize that overall cultural alienation demonstrates positive associations with psychological distress, manifesting in elevated symptoms of depression, anxiety, and related emotional difficulties. Moreover, building on Berry and Sam’s (1997) acculturation model and belongingness theory (Baumeister and Leary, 1995), which emphasize the fundamental role of social bonds in successful adaptation, we propose that cultural loneliness exerts particularly strong effects on mental health outcomes. Specifically, we expect the impact of cultural loneliness to surpass the influence of cultural separation, sense of disharmony, and perceived discrimination, as the absence of meaningful social connections may fundamentally undermine psychological well-being regardless of other adaptation challenges. Furthermore, we anticipate that examining these dimensions through both variable-centered and person-centered approaches will reveal distinct alienation profiles, with students demonstrating elevated levels across multiple dimensions experiencing more severe psychological distress. Although demographic factors such as gender or language background may influence these patterns, we expect alienation constructs to demonstrate stronger explanatory power. Through this investigation, we aim to elucidate how cultural alienation’s various dimensions shape minority students’ psychological experiences, ultimately informing the development of evidence-based interventions that promote both academic success and psychological well-being in this vulnerable population.

## Methods

### Participants and procedure

A total of 73 minority pre-college students of Mongolian ethnicity were recruited from three universities in Inner Mongolia’s major cities (Hohhot, Baotou, and Hulunbuir). All participants were enrolled in minority preparatory programs designed to facilitate their transition to university-level education. The sample comprised 23 males and 50 females, with 67 participants (21 males, 46 females) reporting Mongolian as their primary language of instruction and daily communication, while 6 participants (2 males, 4 females) identified Chinese as their dominant language.

Data collection took place in classroom settings during scheduled academic periods. Following an overview of the study objectives and obtaining written informed consent, participants completed a battery of self-report measures, including the SCL-90 and the Cultural Alienation Scale. All instruments were administered in Chinese under standardized instructions, with completion time averaging 20 min. Research assistants, trained in survey administration, were present throughout to address questions and ensure participant privacy. The study protocol received approval from the institutional ethics committee. Participants received course credit or a debriefing session for their involvement, with no additional incentives provided.

### Measures

Symptom Checklist–90 (SCL-90). Psychological distress was measured using the 90-item Symptom Checklist–90 (SCL–90; Derogatis et al., 1976), a widely used self-report inventory assessing nine domains of psychopathology (e.g., Somatization, Depression, Anxiety, Hostility). We utilized a validated Chinese-language version of the SCL-90 (Shulin and Linjiang, 2003) that has demonstrated robust psychometric properties among Chinese populations. Each item is rated on a 5-point scale from 1 (“not at all”) to 5 (“extremely”), indicating symptom severity over the past week. Extensive validation studies have documented strong internal consistency with Cronbach’s *α* values typically exceeding 0.80 for both the total scale and subscales in Chinese samples (Derogatis et al., 1976; Shulin and Linjiang, 2003), along with test–retest correlations above 0.70. In the current study, we computed scores for all nine clinical dimensions and an overall mean (SCL-90 total) representing general psychological distress.

Cultural Alienation Scale. Participants completed a 37-item Cultural Alienation Scale specifically developed to assess cultural experiences of minority students in predominantly Han Chinese educational contexts (Xi and Dong, 2005). The instrument measures four distinct dimensions: Cultural Loneliness (15 items; e.g., “I feel distanced when studying and living in Han-dominant schools”; “I often feel lonely in Han-dominant areas”), Cultural Separation (11 items), Sense of Disharmony (5 items; e.g., “I cannot understand many interpersonal principles in Han culture”; “I cannot agree with Han people’s standards of beauty”), and Perceived Discrimination (6 items). Items are rated on a 5-point scale ranging from 1 (“strongly disagree”) to 5 (“strongly agree”), with higher scores reflecting greater perceived cultural alienation. Psychometric evaluation in Chinese minority student samples has demonstrated excellent internal consistency (Cronbach’s *α* = 0.92) for the total scale, with subscale α values consistently above 0.70 (Xi and Dong, 2005). The hypothesized four-factor structure has been supported through confirmatory factor analyses. For the present study, we calculated mean scores for each subscale and an overall cultural alienation score by averaging across all items.

### Data analysis strategy

All statistical analyses were conducted using Python (version 3.8). Data management and preprocessing were performed using pandas (McKinney, 2010). Missing values in the SCL-90 and cultural alienation questionnaires were addressed through linear interpolation to preserve data integrity. We computed factor scores for SCL-90 using established 9-factor assignments (Derogatis et al., 1976) and calculated mean scores across the four cultural alienation dimensions (Cultural Loneliness, Cultural Separation, Sense of Disharmony, and Perceived Discrimination).

The analysis began with descriptive statistics for all SCL-90 factors and cultural alienation dimensions, followed by Pearson correlation analyses to examine bivariate relationships among key variables. Although the SCL-90 comprises nine subscales, we subsequently focused our regression and clustering analyses on the total score and four specific subscales—Interpersonal Sensitivity, Depression, Anxiety, and Hostility. These dimensions were selected due to their theoretical and empirical relevance to cross-cultural adaptation, especially regarding the emotional and relational challenges minority students often encounter when negotiating new cultural environments. Other subscales were analyzed descriptively but excluded from primary models to maintain theoretical parsimony and address our principal research objectives. Using the statsmodels package in Python (Seabold and Perktold, 2010), we constructed multiple linear regression models to investigate the unique contributions of cultural alienation dimensions in predicting SCL-90 total score and the selected subscales, while controlling for gender and primary language of instruction. Multicollinearity among the four cultural alienation dimensions was assessed using variance inflation factors (VIF). Model assumptions were verified through both visual inspection and numerical diagnostics.

To complement our variable-centered approach, we employed k-means clustering on the four cultural alienation dimensions. To evaluate cluster stability, we computed silhouette coefficients for two-, three-, and four-cluster solutions. The two-cluster solution was selected based on the highest silhouette coefficient and theoretical alignment with high versus low alienation profiles. These distinct cultural alienation profiles were then compared using one-way ANOVAs to examine differences in SCL-90 total and key subscale scores. Statistical significance was established at *p* < 0.05. All results are presented with appropriate confidence intervals where applicable.

## Results

Descriptive analyses revealed that participants’ overall cultural alienation averaged 2.40 (*SD* = 0.48), falling notably below the theoretical midpoint of 3.00. According to Xi and Dong’s (2005) interpretive guidelines, this mean value suggests a relatively modest level of cultural alienation within our sample. Examination of the four constituent dimensions revealed a consistent pattern: Cultural Loneliness (*M* = 2.38), Cultural Separation (*M* = 2.19), Sense of Disharmony (*M* = 2.76), and Perceived Discrimination (*M* = 2.52), with all dimensions remaining below the midpoint threshold.

Analysis of psychological distress via the SCL-90 yielded an overall total score of 1.47 (*SD* = 0.44), indicating moderate general psychological distress. The subscales of primary interest demonstrated consistent patterns: Interpersonal Sensitivity (*M* = 1.57), Depression (*M* = 1.50), Anxiety (*M* = 1.43), and Hostility (*M* = 1.45), with all means falling appreciably below the clinical threshold of 2.00 (Table 1). These findings indicate that participants reported neither pronounced cultural alienation nor significant psychological distress.

**Table 1:** Descriptive statistics for SCL–90 factor scores among Mongolian minority pre-college students.

Factor	Mean	Std
Somatization	1.308	0.394
Obsessive-compulsive	1.731	0.553
Interpersonal sensitivity	1.567	0.527
Depression	1.502	0.46
Anxiety	1.429	0.541
Hostility	1.454	0.551
Phobic anxiety	1.415	0.486
Paranoid ideation	1.434	0.481
Psychoticism	1.381	0.531
Other	1.464	0.478
Total	1.469	0.438

**Figure 1:**
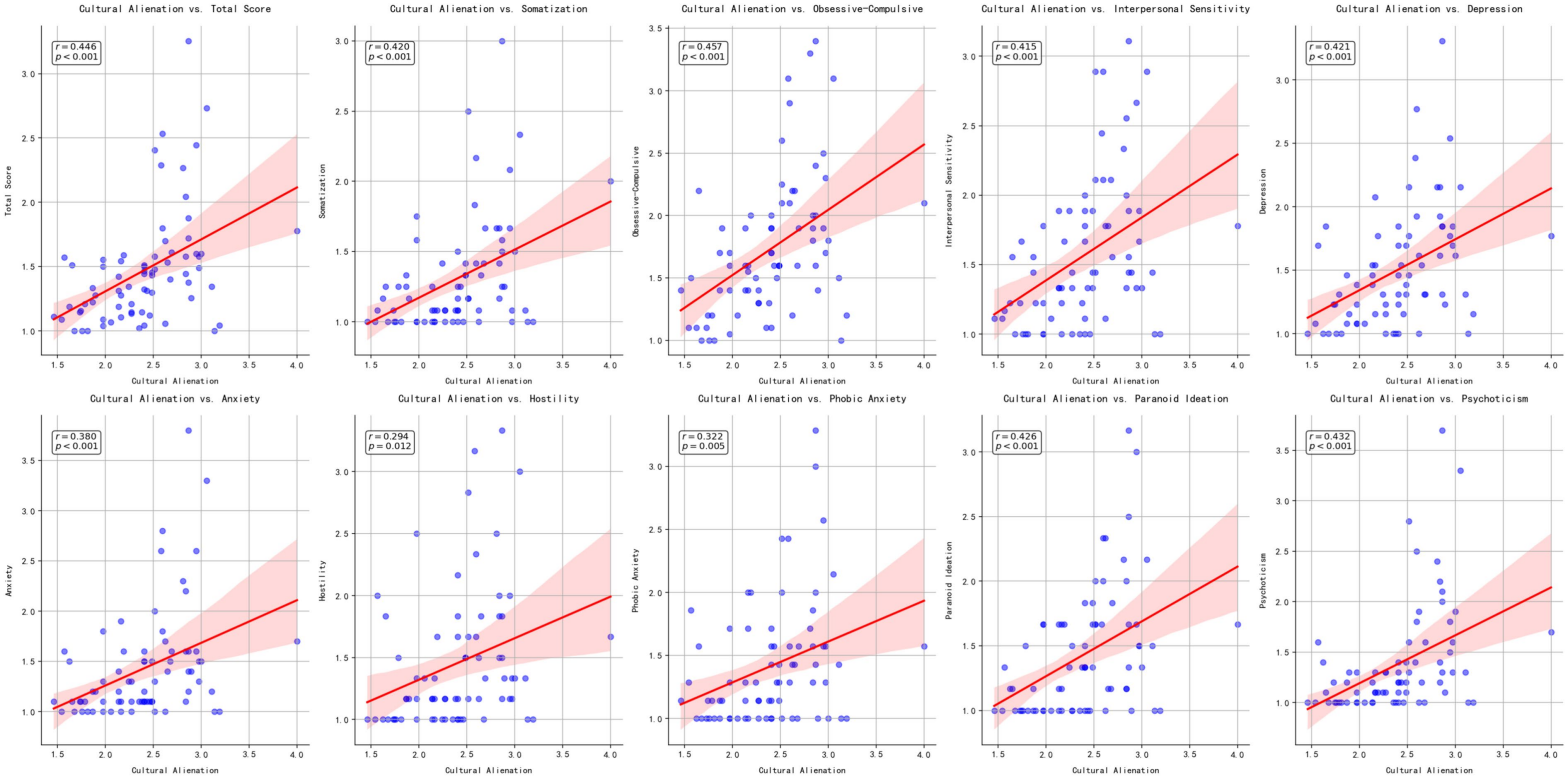
Scatter plots illustrating the associations between overall cultural alienation and the SCL–90 total and subscale scores, with lines of best fit and 95% confidence intervals.

Following these initial descriptive analyses, we examined whether participants’ overall cultural alienation was associated with the SCL-90 total score and its subscales. Pearson correlations showed that cultural alienation correlated moderately with the SCL-90 total score (*r* = 0.446, *p* < 0.001) and several key dimensions, including Obsessive-Compulsive (*r* = 0.457, *p* < 0.001) and Paranoid Ideation (*r* = 0.426, *p* < 0.001; see Figure 1). While most subscales demonstrated significant positive associations with cultural alienation, Hostility (*r* = 0.294, *p* = 0.012) and Phobic Anxiety (*r* = 0.322, *p* = 0.005) were relatively weaker, and Other showed a modest link (*r* = 0.272, *p* = 0.020). These patterns suggest that higher cultural alienation aligns with a greater degree of psychological distress among participants.

Next, we separately investigated each of the four cultural alienation dimensions—Cultural Loneliness, Cultural Separation, Sense of Disharmony, and Perceived Discrimination—in relation to SCL-90. Cultural Loneliness displayed the strongest and most consistent correlations, notably with Depression (*r* = 0.506, *p* < 0.001), Anxiety (*r* = 0.474, *p* < 0.001), and the SCL-90 total score (*r* = 0.501, *p* < 0.001). Cultural Separation was modestly related to several SCL-90 factors—for instance, Somatization (*r* = 0.321, *p* = 0.006) and Obsessive-Compulsive (*r* = 0.276, *p* = 0.018)—yet did not reach significance for Anxiety (*p* = 0.112) or Hostility (*p* = 0.230). Sense of Disharmony evidenced fewer robust associations, correlating with Obsessive-Compulsive (*r* = 0.250, *p* = 0.033) and Paranoid Ideation (*r* = 0.231, *p* = 0.049), but not with the total SCL-90 (*p* = 0.198). In contrast, Perceived Discrimination demonstrated moderate associations across multiple SCL-90 subscales, including Somatization (*r* = 0.395, *p* < 0.001) and Interpersonal Sensitivity (*r* = 0.390, *p* < 0.001), suggesting that feeling discriminated against relates to a general elevation in symptom reporting.

**Figure 2:**
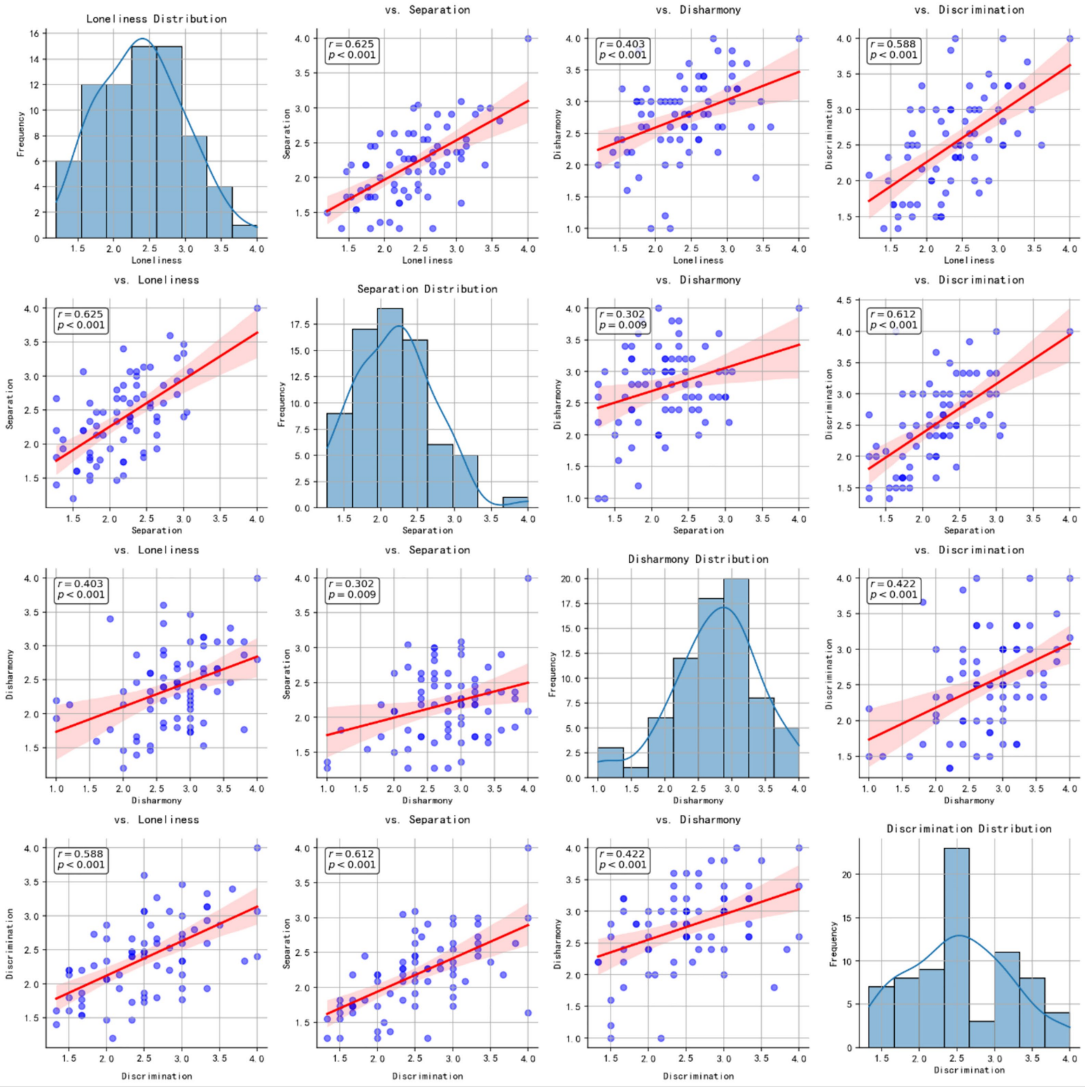
Distributions and pairwise correlations of the four cultural alienation dimensions (cultural loneliness, cultural separation, sense of disharmony, and perceived discrimination), with lines of best fit and 95% confidence intervals.

We also explored intercorrelations among the four cultural alienation dimensions to assess their overlap (see Figure 2). Cultural Loneliness and Cultural Separation were highly correlated (*r* = 0.625, *p* < 0.001), and both showed strong links to Perceived Discrimination (*r*s = 0.588–0.612, *p*s < 0.001). Sense of Disharmony was moderately associated with the other three dimensions (e.g., *r* = 0.403 with Cultural Loneliness, *p* < 0.001), suggesting it partially overlaps but remains somewhat distinct.

**Figure 3:**
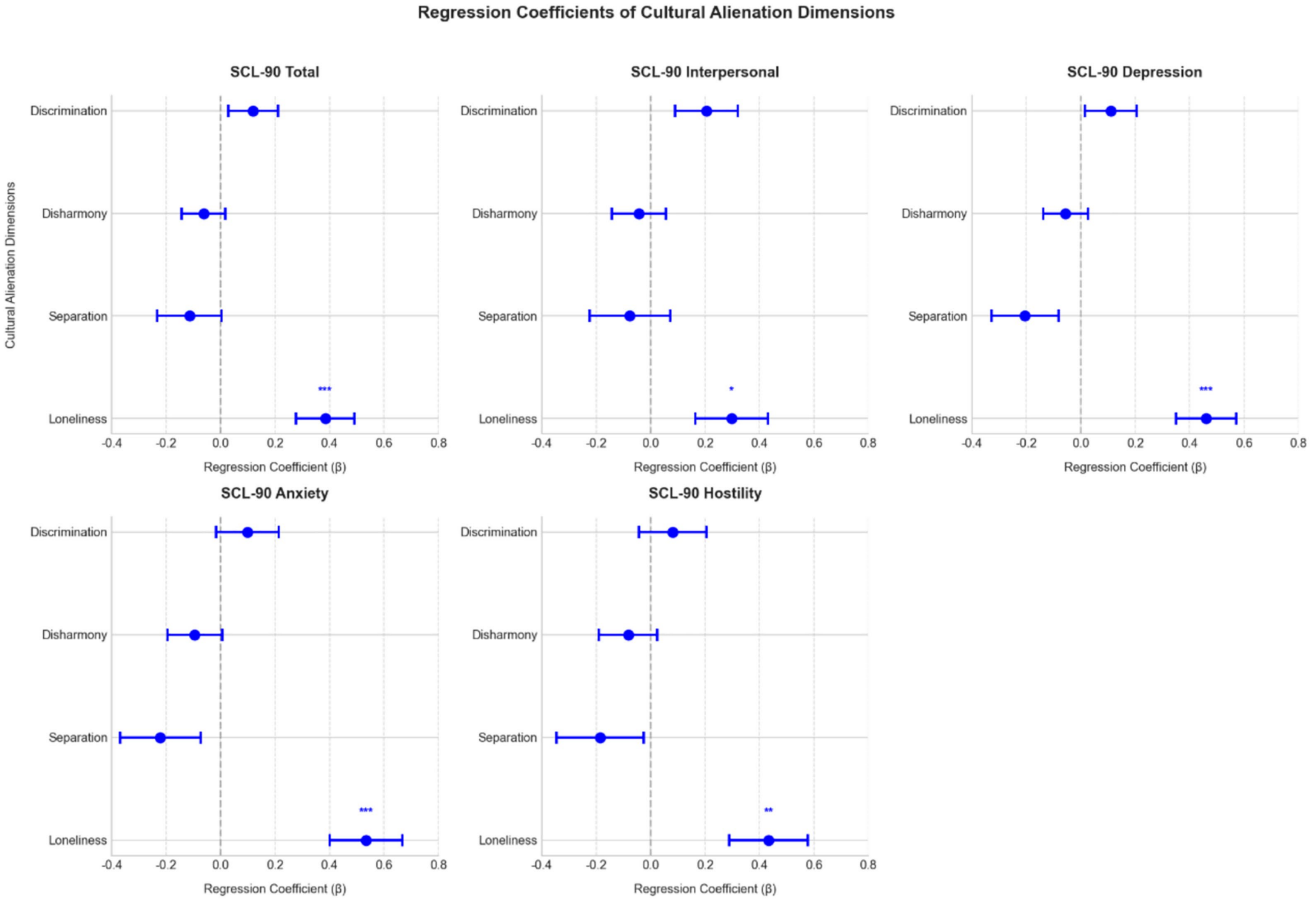
Regression coefficients for cultural alienation dimensions in predicting SCL–90 indices. Each plot displays the regression coefficients (*β*) of cultural loneliness, cultural separation, sense of disharmony, and perceived discrimination (with error bars representing 95% confidence intervals) for one of the five SCL–90 outcomes (total, interpersonal sensitivity, depression, anxiety, and hostility). *p < 0.05, **p < 0.01, ***p < 0.001.

Building upon these correlational analyses, we conducted separate ordinary least squares (OLS) regressions, using five SCL-90 indices (Total, Interpersonal Sensitivity, Depression, Anxiety, Hostility) as dependent variables. Each model included the four cultural alienation dimensions (Cultural Loneliness, Cultural Separation, Sense of Disharmony, Perceived Discrimination) along with gender and primary language of instruction as control variables. Multicollinearity diagnostics indicated acceptable VIF values for all cultural alienation dimensions (range: 1.28–2.00), confirming that multicollinearity did not bias the regression estimates. Table 2 presents the detailed regression results for SCL-90 Total, Depression, and Anxiety, and Figure 3 visualizes the regression coefficients across all five models. Across these five models, Cultural Loneliness emerged consistently as the most robust predictor. Specifically, Cultural Loneliness significantly predicted SCL-90 Total (*β* = 0.4034, *p* = 0.001), Interpersonal Sensitivity (*β* = 0.2899, *p* = 0.043), Depression (*β* = 0.5050, *p* < 0.001), Anxiety (*β* = 0.5609, *p* < 0.001), and Hostility (*β* = 0.4045, *p* = 0.009). In contrast, the other three dimensions did not reach significance in most models, with only Cultural Separation showing a marginal effect in the Depression model (*p* = 0.057). Neither gender nor language type explained additional variance in psychological distress. The regression model for Depression demonstrated the strongest explanatory power (*R*^2^ = 0.347, *p* < 0.001), while Hostility showed the most modest fit (*R*^2^ = 0.176, *p* = 0.041). These findings suggest that Cultural Loneliness was the primary contributor to psychological distress in this sample, with other dimensions showing less consistent effects.

**Table 2:** Multiple regression results predicting SCL-90 outcomes.

Predictor	SCL-90 total			Depression			Anxiety		
	B	SE	*p*	B	SE	*p*	B	SE	*p*
Cultural loneliness	0.40	0.12	0.001***	0.50	0.12	<0.001***	0.56	0.14	<0.001***
Cultural separation	−0.10	0.12	0.385	−0.21	0.12	0.057	−0.20	0.15	0.175
Sense of disharmony	−0.07	0.08	0.418	−0.06	0.08	0.490	−0.11	0.10	0.297
Perceived discrimination	0.11	0.09	0.252	0.09	0.09	0.348	0.09	0.12	0.453
Gender	0.07	0.10	0.485	0.18	0.10	0.088	0.08	0.13	0.535
Language	0.07	0.17	0.670	0.27	0.17	0.122	−0.08	0.21	0.706
*R*^2^	0.278			0.347			0.260		
*F*	4.24**			5.65***			3.87**		

*p < 0.05, **p < 0.01, ***p < 0.001. Gender: 1 = male, 2 = female. Language: 1 = Chinese, 2 = Mongolian.

Silhouette analysis supported the two-cluster solution (silhouette coefficient = 0.352) over three-cluster (0.259) and four-cluster (0.255) alternatives. Table 3 presents the means and standard deviations for each cultural alienation dimension by cluster. To complement these variable-centered analyses, a K-means clustering analysis (k = 2) based on the four cultural alienation dimensions (Cultural Loneliness, Cultural Separation, Sense of Disharmony, and Perceived Discrimination) revealed two distinct profiles. The first cluster demonstrated elevated cultural alienation across all dimensions (Cultural Loneliness = 2.69, Cultural Separation = 2.45, Sense of Disharmony = 3.00, Perceived Discrimination = 2.95), while the second cluster showed markedly lower scores (Cultural Loneliness = 1.93, Cultural Separation = 1.78, Sense of Disharmony = 2.39, Perceived Discrimination = 1.88). To examine the psychological correlates of these profiles, we conducted one-way ANOVAs on five SCL-90 indicators: Total score, Interpersonal Sensitivity, Depression, Anxiety, and Hostility.

**Table 3:** Means and standard deviations of cultural alienation dimensions by cluster.

Dimension	High-alienation (*n* = 31)	Low-alienation (*n* = 42)
	M (SD)	M (SD)
Cultural loneliness	2.69 (0.48)	1.93 (0.41)
Cultural separation	2.45 (0.47)	1.78 (0.44)
Sense of disharmony	3.00 (0.55)	2.39 (0.59)
Perceived discrimination	2.95 (0.46)	1.88 (0.47)

**Figure 4:**
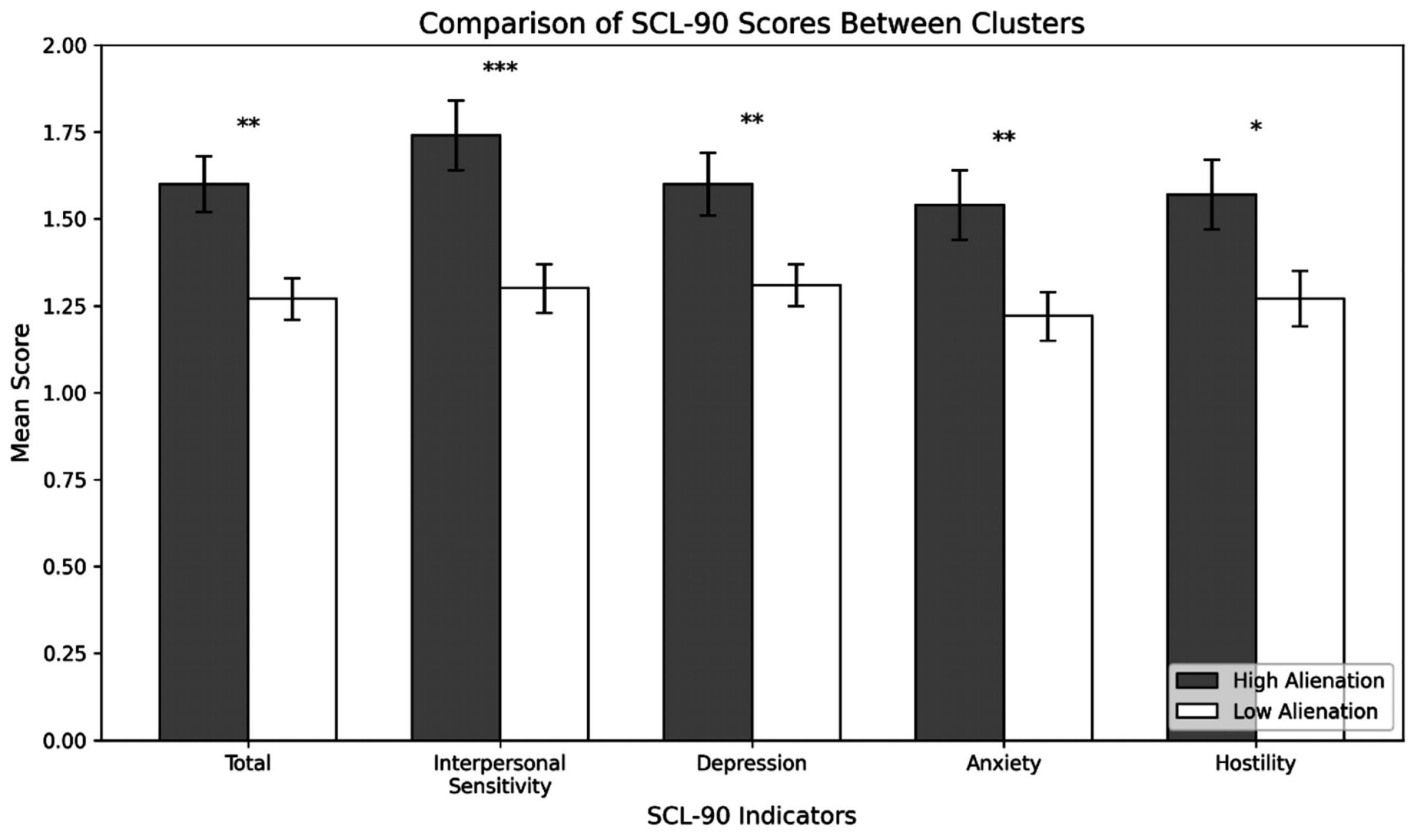
Comparison of mean SCL–90 scores between the high- and low-alienation clusters. Error bars represent standard errors. *p < 0.05, **p < 0.01, ***p < 0.001.

The analyses revealed systematic differences between clusters across all SCL-90 variables, with the high-alienation cluster consistently reporting greater psychological distress than the low-alienation cluster (see Figure 4). The high-alienation group showed an elevated SCL-90 total score (*M* = 1.60) compared to the low-alienation group (*M* = 1.27), *F*(1, 71) = 11.57, *p* < 0.001, η^2^ = 0.14. This pattern was particularly pronounced for Interpersonal Sensitivity (1.74 vs. 1.30, *F*(1, 71) = 14.09, *p* < 0.001, η^2^ = 0.17). Similar differences emerged for Depression, Anxiety, and Hostility, with the high-alienation cluster showing significantly higher scores across all measures (*p*s < 0.05). These findings demonstrate that individuals characterized by higher levels of cultural alienation reported greater psychological distress across multiple domains compared to those with lower alienation profiles.

## Discussion

This investigation examined the complex interplay between cultural alienation and psychological adaptation among minority students navigating the transition to higher education. Given the cross-sectional nature of our data, the following interpretations are guided by theoretical frameworks (Berry’s acculturation model and belongingness theory) rather than causal claims. Through a multifaceted analysis of cultural alienation—encompassing experiences of cultural loneliness, separation, disharmony, and perceived discrimination—we sought to understand how these distinct yet interrelated dimensions contribute to students’ psychological well-being during a critical developmental period. Our investigation centered on three primary objectives: evaluating the association between overall cultural alienation and psychological distress, identifying the most salient alienation dimensions predictive of specific SCL–90 subscales, and exploring whether distinct cultural alienation profiles correspond to varying levels of psychological symptomatology. Our sample comprised minority pre-college students attending universities where the institutional and social contexts predominantly reflect Han Chinese cultural norms and practices. The results revealed that while participants’ overall cultural alienation levels were moderate, they nevertheless showed meaningful associations with psychological distress. Cultural loneliness emerged as the most robust predictor of SCL–90 scores among the four alienation dimensions, consistently overshadowing the effects of cultural separation, sense of disharmony, and perceived discrimination. Moreover, cluster analysis identified a distinct high-alienation subgroup that exhibited significantly elevated psychological symptoms compared to their low-alienation peers.

Our analyses revealed significant associations between overall cultural alienation and multiple facets of psychological functioning. From a cross-cultural adaptation perspective, even moderate levels of alienation can generate psychological strain when minority students feel disconnected from the dominant culture. The correlation patterns showed particularly strong links between alienation and specific SCL-90 dimensions, notably obsessive-compulsive tendencies and paranoid ideation, possibly reflecting the cognitive burden of chronic cultural misunderstanding. Students who feel persistently out of place might develop heightened vigilance or preoccupation with social interactions. While most SCL-90 dimensions demonstrated significant associations with cultural alienation, several dimensions including hostility, phobic anxiety, and other symptoms showed relatively modest correlations. Additionally, examination of intercorrelations revealed that cultural loneliness, separation, and perceived discrimination were highly interrelated, while sense of disharmony showed more moderate associations with these dimensions. This pattern suggests that sense of disharmony might operate through somewhat different psychological mechanisms compared to the other three dimensions.

Cultural loneliness emerged as the dominant predictor across nearly all SCL-90 indices in our regression analyses. This dimension, capturing profound social isolation and detachment from both mainstream and co-ethnic networks, demonstrated consistent predictive power for total distress and specific subscales—including interpersonal sensitivity, depression, anxiety, and hostility. These findings align with fundamental theories of belongingness and social connection (Baumeister and Leary, 1995; Cacioppo and Hawkley, 2009), suggesting that students experiencing deep cultural loneliness may interpret daily challenges through a lens of personal inadequacy or social exclusion. The pervasive influence of cultural loneliness might reflect its fundamental role in students’ adaptation experiences, as it represents not just the absence of social connections but also potential barriers to establishing meaningful relationships in both host and heritage cultural contexts. This isolation from both cultural spheres may particularly heighten students’ psychological vulnerability during their educational transition. However, the strong association between cultural loneliness and psychological symptoms may partially reflect conceptual proximity between feelings of cultural isolation and general interpersonal distress assessed by the SCL-90. While cultural loneliness specifically captures perceived isolation arising from cultural disconnection, some overlap with SCL-90 interpersonal sensitivity items cannot be ruled out. Future research employing discriminant validity analyses could further clarify the boundaries between these constructs.

The other alienation dimensions—cultural separation, sense of disharmony, and perceived discrimination—showed less consistent relationships with psychological outcomes. Cultural separation’s limited predictive power, particularly in regression models, may reflect its potential to function adaptively when accompanied by strong co-ethnic support, aligning with previous findings on cultural maintenance strategies (Ward and Kennedy, 1999). The notably weak influence of sense of disharmony could indicate either effective institutional policies minimizing overt cultural conflicts or students’ development of coping mechanisms that normalize cultural differences. Similarly, while perceived discrimination showed moderate bivariate correlations with psychological symptoms, its effects diminished in multivariate analyses. This pattern suggests that periodic experiences of discrimination, though challenging, may be less detrimental to mental health than the persistent absence of meaningful social connections, particularly in educational contexts where institutional support mechanisms exist. We speculate that cultural separation may function adaptively when accompanied by strong co-ethnic support networks. However, the present study did not directly measure co-ethnic connections among Mongolian students. Future research should examine whether peer support from fellow minority students moderates or mediates the relationship between cultural separation and psychological outcomes.

Our cluster analysis revealed meaningful patterns in how alienation dimensions combine to influence psychological well-being. The identification of distinct high-alienation and low-alienation profiles suggests that students’ experiences of cultural alienation tend to cluster in systematic ways. Students in the high-alienation cluster consistently reported elevated symptoms across all measured SCL-90 indices, indicating that the co-occurrence of multiple alienation dimensions may intensify psychological distress. This pattern aligns with theoretical frameworks suggesting that cultural adaptation involves multiple, interrelated processes (Berry and Sam, 1997). When students experience concurrent challenges across multiple dimensions—feeling socially isolated while also perceiving discrimination and cultural disharmony—their capacity for effective coping may become overwhelmed, leading to more severe psychological symptoms.

These findings carry important theoretical and practical implications for understanding minority students’ psychological adaptation in transitional educational contexts. The primacy of cultural loneliness in predicting psychological distress suggests that social connection serves as a fundamental mechanism through which other aspects of cultural alienation influence mental health. This aligns with broader theories of human development that emphasize the essential role of social bonds in psychological well-being (Baumeister and Leary, 1995). For educational institutions, these results underscore the need for multifaceted support systems that prioritize social integration while acknowledging the complex interplay of alienation dimensions. While structured mentorship programs and cultural support groups may address immediate social needs, sustainable interventions should also consider how institutional policies and practices might inadvertently contribute to or mitigate different forms of cultural alienation. Importantly, institutional structures may differentially mitigate the four alienation dimensions. For instance, peer mentoring programs and cultural student organizations may primarily address cultural loneliness by fostering meaningful social connections, while bilingual academic support services may reduce cultural separation. Dormitory assignments that facilitate interaction between minority and majority students could potentially influence perceived discrimination and sense of disharmony. Future intervention research should examine how specific institutional mechanisms differentially affect each alienation dimension. Additionally, emerging research suggests that mobile applications can effectively enhance campus support services for university students (Subbarao et al., 2025), potentially offering scalable digital platforms for delivering psychological support and fostering social connections among minority students. At the individual level, cognitive restructuring techniques may help minority students transform negative thoughts arising from cultural loneliness into more adaptive cognitions (Takdir et al., 2025), providing a structured therapeutic approach to address the psychological consequences of cultural alienation.

Several methodological considerations warrant attention when interpreting these findings. Our sample size, though providing sufficient statistical power for primary analyses, suggests the need for cautious interpretation of more complex statistical models. Additionally, our sample was drawn from three urban institutions in Inner Mongolia, which may limit generalizability to minority students in rural areas or other regions of China where institutional resources and cultural dynamics may differ. Moreover, the cross-sectional nature of our data limits causal inferences about the relationship between alienation and psychological distress. While our theoretical framework suggests that alienation contributes to psychological symptoms, the possibility of bidirectional influences cannot be ruled out. The Cultural Alienation Scale, although contextually appropriate for our sample, may capture cultural experiences specific to the Chinese educational environment, potentially affecting comparisons with other cultural contexts.

Future research should build upon these findings to further elucidate the mechanisms through which cultural alienation influences psychological adaptation. Specifically, studies might explore how different configurations of alienation dimensions interact with institutional and personal resources to shape adaptation outcomes. Longitudinal investigations could help clarify the temporal dynamics between alienation experiences and psychological symptoms, particularly during critical transition periods in minority students’ academic trajectories. Given our findings regarding cultural loneliness’s central role, research examining how social networks develop and function in these educational contexts could be especially valuable. Such work might consider how students’ connections with both heritage and host culture peers influence their psychological adaptation, and how institutional structures either facilitate or impede the development of meaningful social bonds. Moreover, investigation of potential protective factors—such as bicultural competence or institutional support systems—could help identify specific intervention targets for supporting minority students’ psychological well-being. Recent research has identified psychological traits including resilience and self-efficacy as significant predictors of positive outcomes among university students (Shabbir et al., 2025), suggesting that future studies might explore how these characteristics buffer against the negative effects of cultural alienation among minority student populations.

In conclusion, this study advances our understanding of how cultural adaptation processes shape minority students’ psychological experiences during critical educational transitions. By demonstrating the particular salience of cultural loneliness and revealing distinct patterns of alienation experiences, our findings contribute to both theoretical models of cultural adaptation and practical approaches to supporting minority students. The results suggest that successful adaptation involves more than simply acquiring academic or linguistic competencies; it requires careful attention to students’ social–emotional experiences and the complex ways in which different forms of cultural alienation interact to influence psychological well-being. As institutions continue to develop support systems for minority students, understanding these nuanced patterns of cultural alienation and their psychological implications becomes increasingly crucial for fostering inclusive and effective educational environments.

## Data Availability

The raw data supporting the conclusions of this article will be made available by the authors, without undue reservation.
